# Enhancement of cytotoxicity and induction of apoptosis by cationic nano-liposome formulation of *n*-butylidenephthalide in breast cancer cells

**DOI:** 10.7150/ijms.51439

**Published:** 2021-06-05

**Authors:** Xiao-Fan Huang, Kai-Fu Chang, Yu-Ling Lin, Kuang-Wen Liao, Chih-Yen Hsiao, Gwo-Tarng Sheu, Nu-Man Tsai

**Affiliations:** 1 Institute of Medicine, Chung Shan Medical University, Taichung, 40201, Taiwan, ROC.; 2 Department of Medical Laboratory and Biotechnology, Chung Shan Medical University, Taichung, 40201, Taiwan, ROC.; 3 Agricultural Biotechnology Research Center, Academia Sinica, Taipei, 11529, Taiwan, ROC.; 4 Department of Biological Science and Technology, National Chiao Tung University, Hsinchu, 30010, Taiwan, ROC.; 5 Institute of Molecular Medicine and Bioengineering, National Chiao Tung University, Hsinchu, 30010, Taiwan, ROC.; 6 Division of Nephrology, Department of Internal Medicine, Ditmanson Medical Foundation Chia-Yi Christian Hospital, Chia-Yi, 60002, Taiwan, ROC.; 7 Department of Hospital and Health Care Administration, Chia Nan University of Pharmacy and Science, Tainan, 71710, Taiwan, ROC.; 8 Clinical Laboratory, Chung Shan Medical University Hospital, Taichung, 40201, Taiwan, ROC.

**Keywords:** Polycationic liposome containing PEI and polyethylene glycol complex (LPPC), n-Butylidenephthalide (BP), Cell apoptosis, Cell cycle, Synergistic effect

## Abstract

Breast cancer is the second most common malignancy in women. Current clinical therapy for breast cancer has many disadvantages, including metastasis, recurrence, and poor quality of life. Furthermore, it is necessary to find a new therapeutic drug for breast cancer patients to meet clinical demand. n-Butylidenephthalide (BP) is a natural and hydrophobic compound that can inhibit several tumors. However, BP is unstable in aqueous or protein-rich environments, which reduces the activity of BP. Therefore, we used an LPPC (Lipo-PEG-PEI complex) that can encapsulate both hydrophobic and hydrophilic compounds to improve the limitation of BP. The purpose of this study is to investigate the anti-tumor mechanisms of BP and BP/LPPC and further test the efficacy of BP encapsulated by LPPC on SK-BR-3 cells. BP inhibited breast cancer cell growth, and LPPC encapsulation (BP/LPPC complex) enhanced the cytotoxicity on breast cancer by stabilizing the BP activity and offering endocytic pathways. Additionally, BP and LPPC-encapsulated BP induced cell cycle arrest at the G_0_/G_1_ phase and might trigger both extrinsic as well as intrinsic cell apoptosis pathway, resulting in cell death. Moreover, the BP/LPPC complex had a synergistic effect with doxorubicin of enhancing the inhibitory effect on breast cancer cells. Consequently, LPPC-encapsulated BP could improve the anti-cancer effects on breast cancer *in vitro*. In conclusion, BP exhibited an anti-cancer effect on breast cancer cells, and LPPC encapsulation efficiently improved the cytotoxicity of BP via an acceleration of entrapment efficiency to induce cell cycle block and apoptosis. Furthermore, BP/LPPC exhibited a synergistic effect in combination with doxorubicin.

## Introduction

Breast cancer is the second most common malignancy in women and causes more than a half-million deaths each year worldwide 1. Additionally, the incidence of breast cancer continues to increase despite the progress against malignancy 2. Unfortunately, some problems remain after cancer therapy, such as metastasis, recurrence, and poor quality of life. Thus, instead of killing the cancer cells, the latest concept for cancer treatment is to prevent carcinogenesis at an early stage or after therapy, which is called chemoprevention and is becoming a new trend 3, 4. Chemo-preventive agents are able to inhibit, delay or reverse premalignant lesions or carcinogenesis and include soy products, a promising chemo-preventive option for breast cancer 5-8, and resveratrol, an anti-cancer agent against breast cancer 9, 10.

*Angelica sinensis* radix, known as dong-quai, is commonly used in traditional Chinese medicine, and is mainly applied for anemia and gynecological dysfunction in traditional applications. n-Butylidenephthalide (BP), a natural compound extracted from *Angelica sinensis* 11, 12, exhibits various pharmacological activities, such as alleviating liver injury and fibrosis 13, acaricidal activities 14, and neuroprotective activities 15-17. It is widely reported in the literature that BP induces cell cycle arrest or cell apoptosis in various human cancer cell lines *in vitro* or *in vivo*, including brain, lung, colon, prostate, breast, and liver cancer 18-25. BP reduces cell cycle regulators, suppresses the activity of telomerase reverse transcriptase, and activates the Nur77-mediated apoptosis pathway, causing tumor cell apoptosis 26, 27. In view of the good therapeutic effects of BP, however, the anti-cancer effects of BP and BP/LPPC on SK-BR-3 cells have yet to be elucidated. To date, the clinical application of BP has been limited because of its low stability and solubility in aqueous solutions. In water solutions, BP usually forms a dimer, being hydrated, or oxidized. Moreover, the major limitation of BP is rapid metabolism and elimination through urine or stool 27. To conquer these problems, using a drug carrier would be a good way to improve the bioavailability of BP. Therefore, the delivery of the anti-cancer drug into cancer cells via nanoparticles may be an advancement in cancer therapy.

Liposome complex containing PEI and PEG (Lipo-PEG-PEI complex, LPPC) encapsulation, a new type drug carrier, has been verified the encapsulation efficacy of LPPC on curcumin, doxorubicin, and BP in breast, colon, glioma, and melanoma cancer therapy *in vitro* and *in vivo* and suggests that LPPC could be used as an effective drug carrier for stabilizing the activity of drugs 28-36. LPPC is composed of DOPC and DLPC, which are two natural lipids that can be metabolized by the liver. The LPPC particle is sphere-shaped with an average diameter of 180 nm and surrounded by electron-dense materials which is detected about 40.1 ± 9.5 mV by Zetasizer under transmission electron microscopy 28. Moreover, in our previous study, we have demonstrated that the particle sizes of BP/LPPC is about 200 nm to 280 nm with 38 mV and its maximal encapsulation capacity is about 830 µg in 1 mg of LPPC. The rate of BP release from BP/LPPC is 6%-13% at 4 °C in H_2_O and is greater than 70% at 37 °C after 4 days in protein-rich solution 29. Furthermore, researchers have proposed that a nanocarrier can improve the efficacy of drug delivery through internalized pathways, such as phagocytosis, or other endocytic pathways, including clathrin-mediated, caveolae-mediated, clathrin or caveolae independent and macropinocytosis 37, 38.

Therefore, the purpose of this study is to elucidate the anti-tumor mechanisms of BP and further test the efficacy of the drug carrier - BP encapsulated by LPPC *in vitro*. The results of this study demonstrated that BP inhibited the growth of breast cancer cells. BP encapsulated by LPPC improved the stability in a water solution and protein-rich environment at 4 °C and 37 °C. Additionally, LPPC enhanced the cytotoxicity of BP on breast cancer through stabilizing the BP activity and offering endocytic pathways, including clathrin-mediated, caveolae-mediated, and clathrin or caveolae independent pathways. BP and BP/LPPC both induced cell cycle arrest and apoptosis; however, compared to BP alone, BP/LPPC provided a faster way to inhibit cell cycle regulators, cause cell cycle arrest and activate the caspase cascade, resulting in cell apoptosis. Moreover, BP/LPPC also had a synergistic effect with the clinical drug doxorubicin. Therefore, this study demonstrated that LPPC loaded with BP displayed antiproliferative and apoptotic activities in breast cancer cells.

## Material and methods

### Chemicals and reagents

LPPC and liposomal BP (BP/Lipo) were provided by the lab of Dr. Kuang-Wen Liao, National Chiao Tung University, Hsinchu, Taiwan. BP was purchased from Lancaster Synthesis (Newgate, Morecambe, UK). PEG-1500 and BCA Protein Assay Reagent were purchased from Thermo Fisher Scientific (Waltham, MA, USA). Phenol (chloroform, 2× RIPA lysis buffer and protease inhibitor) was purchased from Bio Basic Inc. (Toronto, Canada). MTT, amiloride hydrochloride hydrate (AHH), filipin III (FIII), chlorpromazine hydrochloride (CPZ), propidium iodide (PI), RNAse A, dimethyl sulfoxide (DMSO) and *in situ* cell death detection kit fluorescein were purchased from Sigma-Aldrich Chemicals Co. (St. Louis, MO, USA). A T-Pro LumiFast plus Chemiluminescence Detection Kit was purchased from T-Pro Biotechnology (New Taipei County, Taiwan). Dulbecco's Modified Eagle's Medium (DMEM), Roswell Park Memorial Institute (RPMI) 1640 Medium, fetal bovine serum (FBS), HEPES buffer solution, sodium pyruvate (100 mM), and 0.05% trypsin-EDTA were purchased from Gibco (Grand Island, NY, USA).

### Cell culture

SK-BR-3, MDA-MB-231, MCF-7, BCM-1, CMT-1, JC, CF-41, and SVEC cell lines were purchased from American Type Culture Collection (Manassas, VA, USA) and maintained according to the supplier's recommendations. Cell culture growth media using DMEM or RPMI-1640 were supplemented with 10% heat-inactivated fetal bovine serum, 1% HEPES buffer solution, 1% sodium pyruvate and 1% penicillin/streptomycin. All cells were observed daily under microscopy and subcultured with 0.05% trypsin-EDTA. For MDA-MB-231 and MCF-7 cells, Femtopath TP53 Exon8 Primer Set, Femtopath EGFR Exon19 and Exon20 Primer Sets, and Femtopath PIK3CA Exon9 Primer Set (HongJing Biotech., New Taipei City, Taiwan) were used to confirm the TP53, EGFR and PIK3CA levels, respectively.

### BP-loaded LPPC preparation

The preparation of BP-loaded LPPC has been previously described 29. Briefly, LPPC was dissolved in distilled/deionized water. After centrifugation, BP (1 mM) was rapidly stirring injected into LPPC (1 mg/ml) in an aqueous solution and incubated for 30 minutes. After centrifugation and supernatant discarding, the mixture was bathed with PEG-1500 (100 mg/ml) and distilled/deionized water for another 30 minutes. Then, the BP-loaded LPPC was collected by centrifugation and stored in a refrigerator at 4 °C for the following experiments. The concentration of BP/LPPC was presented the BP concentration in LPPC encapsulation throughout all experiments.

### Cell proliferation analysis

To evaluate and compare the effect of free BP, BP/Lipo and BP-loaded LPPC on cell viability, breast cancer or normal cells were seeded in 96-well plates at densities of 5×10^3^ or 1×10^4^ per well under 5% CO_2_ at 37 °C overnight. The cells were then exposed to different concentrations of BP, BP/LPPC, BP/Lipo or doxorubicin for 24 and 48 hrs. After incubation, MTT solution (500 μg/ml) was pipetted into each well, and the cells incubated for another 6-8 hrs. The solution was later discarded and dissolved in DMSO. The absorbance was determined using a SpectraMax M5 microplate reader (Molecular Devices; San Jose, CA, USA) at 550 nm, and cell viability was expressed as a percentage of live cells relative to controls. All experiments were performed in triplicate.

### Stability of BP-loaded BP/LPPC

The purpose of the experiments was to evaluate whether LPPC could protect BP activity at 37 °C and protein-rich environment to monitor human physiological state 29. In experimental design, the drugs were divided into four groups: (1) BP dissolved in ddH_2_O at 4 °C (Protein free; storage condition); (2) BP dissolved in 10% FBS at 37 °C (Protein rich; bioactive condition); (3) BP/LPPC dissolved in ddH_2_O at 4 °C (Protein free; storage condition); (4) BP/LPPC dissolved in 10% FBS at 37 °C (Protein-rich; bioactive condition). Each group was administered with 3 mg/ml of BP. All these treatments were incubated for indicated time points (0, 4, 8, and 24 hrs). Data were calculated in IC_50_, and the cytotoxic efficiency was evaluated regarding the stability of BP-loaded BP/LPPC in different environments.

### Determination of BP uptake, amount and endocytotic pathway

To observe the amount of BP entrapment into cells, SK-BR-3 cells (5×10^5^ cells/dish) were seeded on microscope cover glasses (Deckglaser, Germany). Cells were exposed to BP/LPPC or BP (50 μg/ml) and incubated for 0, 15, 30, 45 and 60 minutes. Upon washing with PBS and fixing with 10% neutral formalin, the blue fluorescence of BP was observed and images were acquired via fluorescence microscope (ZEISS AXioskop2; Bremen, Germany) at 400× field. To qualitative the amount of BP entrapping into cells, SK-BR-3 cells (5×10^5^ cells/dish) were seeded in 24-well plates and exposed to BP/LPPC or BP (50 μg/ml), followed by incubation for 0, 15, 30, 45 and 60 minutes. Next, BP was extracted from cells by phenol-chloroform and the BP concentrations of each time point were detected using a full-wavelength fluorescent scanner F-4500 (HITACHI, Tokyo, Japan) at 350 nm. For endocytotic inhibition, SK-BR-3 cells (5×10^5^ cells/dish) were seeded in 24-well plates and exposed to one of the following endocytotic inhibitors for 1 hr: amiloride hydrochloride hydrate (AHH, 13.31 μg/ml), filipin III (FIII, 1 μg/ml) or chlorpromazine hydrochloride (CPZ, 10 μg/ml). Next, we replaced the drug in each well with BP/LPPC or BP (50 μg/ml) and exposed the cells for another 0, 15, 30, 45, 60 and 90 minutes. Then, extracted BP was detected using a full-wavelength fluorescent scanner F-4500 at 350 nm to quantify the amount of BP.

### Analysis of cell cycle distribution

Cells (2×10^6^/10 cm culture plate) were seeded and exposed to BP (60, 90 and 120 μg/ml) or BP/LPPC (20, 40 and 60 μg/ml). The cells were harvested, resuspended, and washed twice with PBS. Cells were incubated overnight at 4 °C with a staining solution that contained propidium iodide (40 μg/ml) and RNase A (100 μg/ml). Samples were analyzed using a FACSCanto II flow cytometer (BD Biosciences, San Jose, CA, USA).

### Detection of cell death

Cells cultured with or without BP (90 μg/ml) for 24 hrs or BP/LPPC (40 μg/ml) for 3 hrs were stained with *in situ* cell death detection kit fluorescein according to the manufacturer's instructions. Cells were washed with PBS, followed by staining with propidium iodide (10 µg/ml) for 10 minutes, and then observed under fluorescence microscopy. The images of typical apoptosis morphology were locally amplified using software Photoshop (Adobe. Photoshop. CS3. Extended v10.0).

### Western blotting assay

The lysate proteins were quantified using BCA Protein Assay Reagent. Each sample load comprised 20 μg total proteins per lane and was resolved by 10-12.5% sodium dodecyl sulfate-polyacrylamide gel electrophoresis. After being transferred onto the polyvinylidene difluoride membrane (PALL; Port Washington, NY, USA), membranes were blocked with 5% skimmed dried milk for 1 hour and incubated overnight with primary antibodies, including anti-pRb, anti-p21, anti-cdk2, anti-cdk4, anti-Fas, anti-Fas-L, anti-procaspase 3, anti-procaspase 8, anti-procaspase 9, anti-Bax, and anti-actin (dilution: 1:200; Santa Cruz Biotechnology Inc, CA, USA); anti-cyclin D1 and anti-cyclin B1 (dilution: 1:1000; iReal Biotechnology Co., Ltd., Hsinchu, Taiwan). The membrane was then washed and incubated with respective anti-mouse, anti-rabbit or anti-goat IgG secondary antibodies followed by horseradish peroxidase, and then protein expression was detected using a T-Pro LumiFast plus Chemiluminescence Detection Kit and the intensity of bands was quantified using ImageJ software (NIH, Bethesda, MD, USA).

### Evaluation of drugs combination effect

Combination studies were determined using MTT assays, and the synergistic effect was evaluated using the combination index (CI) 39, 40. SK-BR-3 and MBA-MD-231 cells were seeded in 96-well plates at a density of 5,000 cells/well. After a 24-hour culture, various conditions were used to treat the cells. Experimental groups included control, doxorubicin (0-1 μg/ml) alone, BP/LPPC (0-60 μg/ml) alone, and a combination of doxorubicin and BP/LPPC treatment. After a 24-hour drug treatment, cell viability was obtained and the CI was calculated using the following formula: [IC_50_ (BP/LPPC+doxorubicin)/IC_50_ (doxorubicin)] + [IC_50_ (BP/LPPC+ doxorubicin)/IC_50_ (BP/LPPC)]. CI values lower than 0.9 indicated synergy, CI values from 0.9 to 1.1 indicated an additive effect, and CI values higher than 1.1 indicated antagonism.

### Statistical analysis

Data obtained were from experiments performed in triplicate and are shown as the mean ± standard deviation (SD). Statistical significance was determined using the unpaired Student's t-test and p < 0.05 was considered as significant.

## Results

### BP inhibited cell proliferation in SK-BR-3 and MDA-MB-231 cells

**Figure 1:**
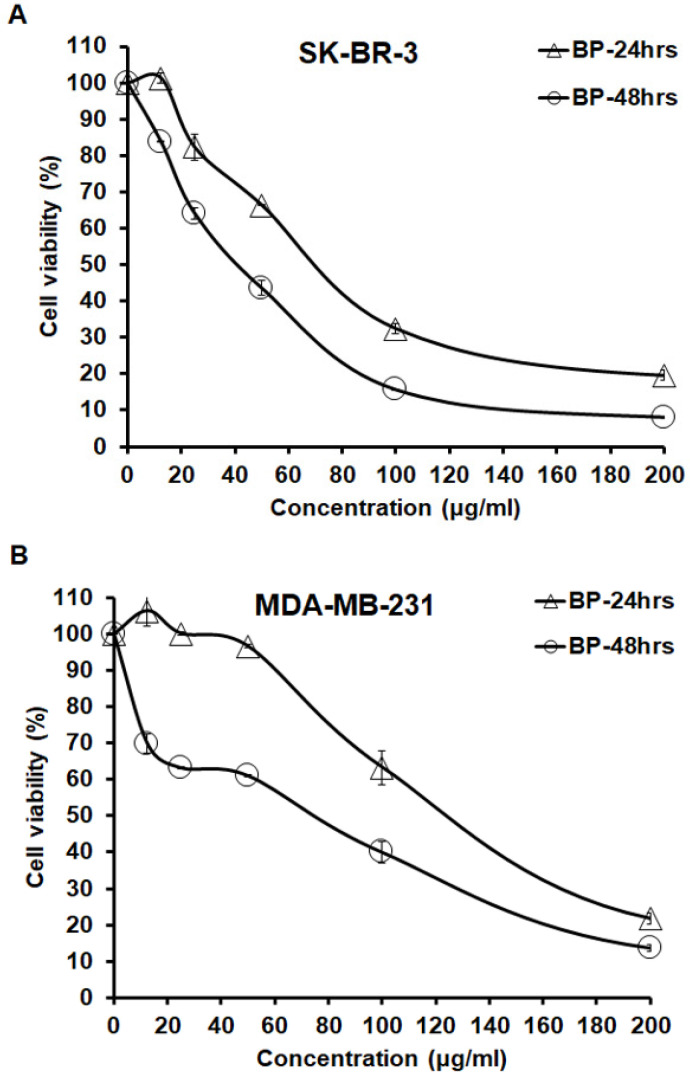
** BP inhibited SK-BR-3 and MDA-MB-231 cells.** SK-BR-3 and MDA-MB-231 cells were separately exposed to BP (0-200 μg/ml) for 24 and 48 hrs. After that, an MTT assay was performed to calculate cell viability; absorbance was measured at 550 nm. (A) SK-BR-3 cells; (B) MDA-MB-231 cells.

The cytotoxicity of BP in human breast cancer cells, triple positive SK-BR-3 cells and negative MDA-MB-231 cells were determined via MTT assay treated with increasing concentrations of BP (0-200 μg/ml) for 24 and 48 hrs. After exposure, BP was effective in reducing the cell viability in a dose- and time-dependent manner. Within 24 hrs of exposure, BP yielded 50% growth inhibition concentration (IC_50_) values of 74.11 ± 0.71 μg/ml and 132.03 ± 3.69 μg/ml in SK-BR-3 and MDA-MB-231 cells, respectively (Table 1). A comparison of IC_50_ values revealed that SK-BR-3 cells were more sensitive to BP than were MDA-MB-231 cells (Figure 1). These results showed that BP effectively inhibited SK-BR-3 and MDA-MB-231 cell growth.

### LPPC encapsulation protected the cytotoxicity of BP and enhanced the cytotoxicity of BP in breast cancer cells

**Figure 2:**
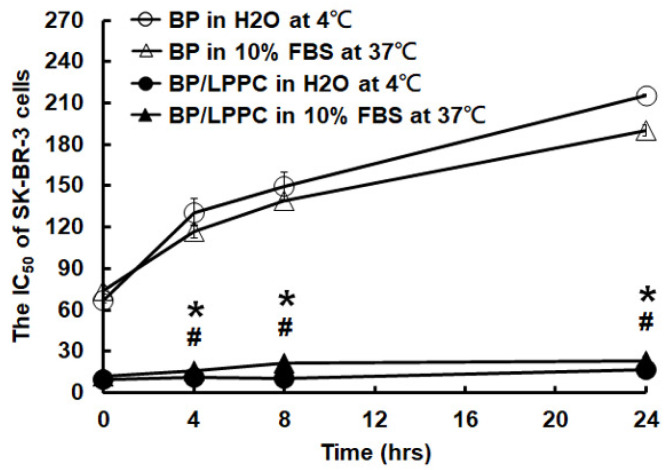
** LPPC protected the activity of BP in different temperatures, as well as in a protein-rich environment.** SK-BR-3 cells were exposed to BP (storage at 4 °C), BP (storage in 10% FBS at 37 °C), BP/LPPC (storage at 4 °C) or BP/LPPC (storage in 10% FBS at 37 °C), and the concentration of 50% inhibition was calculated by MTT assay. *: Indicated a significant difference between BP treatment and BP/LPPC treatment (storage at 4 °C, *P* < 0.05). #: Indicated a significant difference between BP and BP/LPPC (storage in 10% FBS at 37 °C, *P* < 0.05).

**Figure 3:**
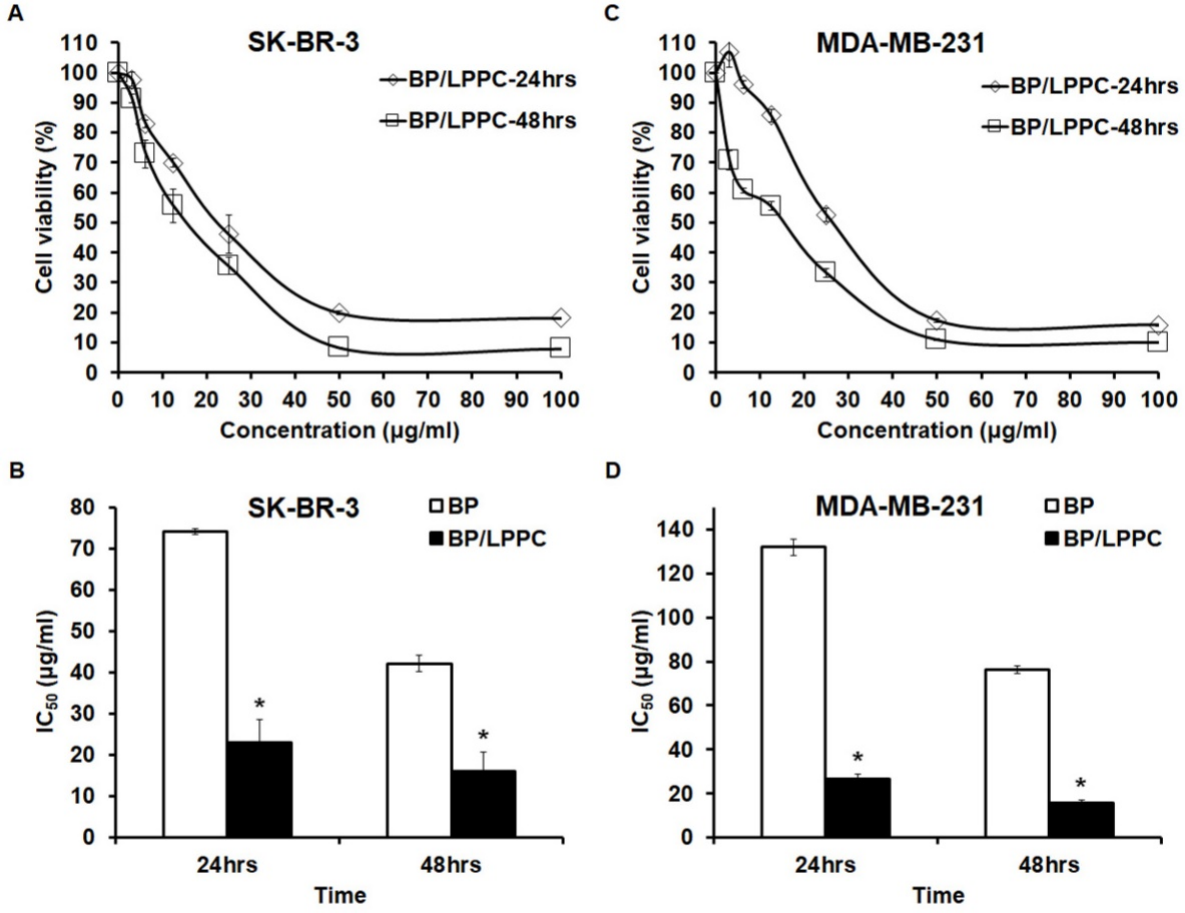
** LPPC encapsulation improved the anti-proliferative ability of BP in breast cancer cell lines.** SK-BR-3 and MDA-MB-231 cells were separately exposed to BP/LPPC (0-100 µg/ml) for 24 and 48 hrs. After that, an MTT assay was performed to calculate cell viability; absorbance was measured at 550 nm. (A, B) SK-BR-3 cells; (C, D) MDA-MB-231 cells. *: Indicated a significant difference between the IC_50_ of BP and the IC_50_ of BP/LPPC (*P* < 0.05).

Previous studies developed by our lab found that LPPC encapsulation protected BP against instability induced by proteins, pH and oxygen in glioblastoma cells. Thus, to further confirm the liposomal BP protection in breast cancer cells, we utilized an MTT assay to test the protective effects of LPPC encapsulation in different environments. The data demonstrated that LPPC encapsulation protected BP against different temperatures, as well as an environment rich in proteins or a water solution, which was similar to prior studies (Figure 2). Next, to examine BP/LPPC-induced cellular inhibitory effects in SK-BR-3 and MDA-MB-231 cells, as shown in Figure 3, both 24- and 48-hrs treatments efficiently reduced the cell viability of breast cancer cells. In addition, both cell types were more sensitive to BP/LPPC-induced cytotoxicity in a dose- and time-dependent manner (Figure 3A and C). Additionally, by comparison, the IC_50_ of BP/LPPC was lower than that of BP, ranging from 3.23- to 4.93-fold in these 2 kinds of cells (Figure 3B and D). These results also indicated that BP encapsulated by LPPC would make breast cancer cells more sensitive to BP treatment. Hence, we further examined the cytotoxic activity of BP and BP/LPPC in other breast cancer and normal cells. The results revealed that LPPC encapsulation indeed enhanced the cytotoxic activity, ranging from 2.60- to 4.93-fold in all seven breast cancer cells. Another drug delivery system was also examined, and the results found that the IC_50_ of BP/LPPC compared with traditional liposomal encapsulation of BP was lower, ranging from 2.85- to 11.6-fold. However, compared to BP/LPPC, doxorubicin was more cytotoxic to breast cancer cells with lower IC_50_ (Table 1). Besides, BP and BP/LPPC both inhibited the growth of SVEC normal cells as well. To compare the selectivity of drugs for tumor and normal cells, the selectivity index (SI) was further calculated, and SI values > 2 were considered as high selectivity to tumor cells 41. As showed in Table 2, the SI was 4.15 in BP alone treatment, 3.23 in BP/LPPC treatment and 1.56 in BP/Lipo treatment, revealing that BP and BP/LPPC exerted good selectivity and BP/Lipo presented poor selectivity. Taken together, LPPC encapsulation indeed protected BP from protein-rich, warm, as well as environments. Moreover, LPPC encapsulation retained the cytotoxic ability of BP and might improve the anti-breast cancer capacity of BP on breast cancer cells.

### LPPC accelerated cellular uptake of BP through endocytic pathway in SK-BR-3 cells

**Figure 4:**
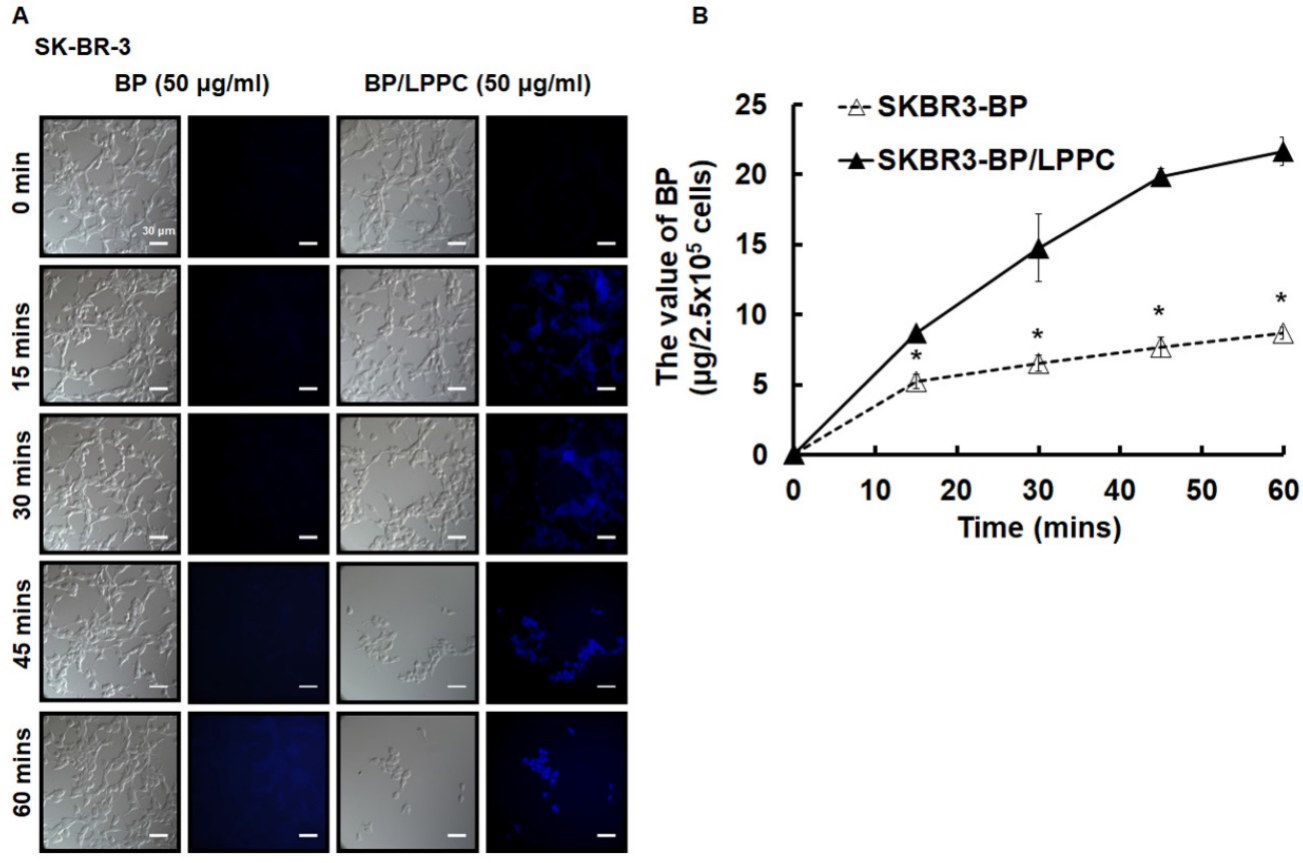
** The amount of BP/LPPC showed rapid uptake by breast cancer cells.** Cells were exposed to BP or BP/LPPC (50 μg/ml) for the indicated time points, and then the fluorescence of BP was observed under microscopy and the content of BP was extracted from phenol-chloroform, followed by detection with absorbance at 350 nm. (A) The amount of BP uptake; (B) The content of BP extracted from cells. *: Indicated a significant difference between BP/LPPC and BP treatment (*P* < 0.05).

**Figure 5:**
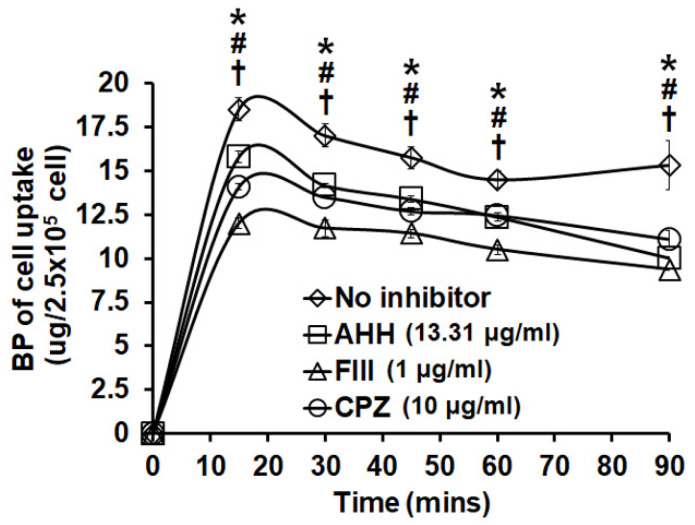
** LPPC encapsulation induced breast cancer cell activation of cell endocytic pathways.** Cells (2.5×10^5^) were pretreated with inhibitors (AHH, FIII and CPZ) for 1 hr. The inhibitors were discarded and cells were exposed to BP/LPPC (50 µg/ml) for the indicated time points, then BP was extracted from phenol-chloroform, followed by detection with absorbance at 350 nm. *, #, †: Indicated a significant difference compared to AHH (*), FIII (#), and CPZ (†) treatment, respectively, with no inhibitors (*P* < 0.05).

We investigated the cellular uptake in SK-BR-3 cells after LPPC encapsulation. Encapsulated BP was observed in the cells within 15 minutes and continuously entered the cells for 60 minutes along with a great amount of cell death. However, nonencapsulated BP was not markedly observed in cells within 60 minutes, and cell death was barely observed after nonencapsulated BP treatment (Figure 4A). According to the above results, the BP content in SK-BR-3 cells was quantified, and the data showed that the BP content continuously increased over 60 minutes with BP/LPPC treatment; however, it did not significantly increase with nonencapsulated BP treatment (Figure 4B). As some studies have shown, liposomes with a positive charge may induce cell endocytosis 37, 38. Hence, we explored the endocytic pathway induced by LPPC in SK-BR-3 cells. The experimental design was to pretreat three endocytic inhibitors, which were included amiloride hydrochloride hydrate (AHH) to inhibit micropinocytosis, filipin III (FIII) to inhibit caveolae dependent pathway and chlorpromazine hydrochloride (CPZ) to inhibit clathrin-dependent pathway, then administered BP/LPPC (40 μg/ml) treatment. The results showed that these three inhibitors reduced the BP content in cells (Figure 5). In contrast, LPPC might have stimulated the activation of these endocytotic pathways to uptake BP. As a result, the data suggested that SK-BR-3 cells were sensitive to LPPC encapsulation-induced cellular uptake. In addition, the results demonstrated that LPPC might activate endocytic pathways to improve the cellular uptake of BP through caveolae-mediated pathways (main), clathrin-dependent (moderate), and micropinocytosis.

### BP/LPPC and BP induced cell cycle arrest at G_0_/G_1_ phase and contributed to apoptosis in SK-BR-3 cells

**Figure 6:**
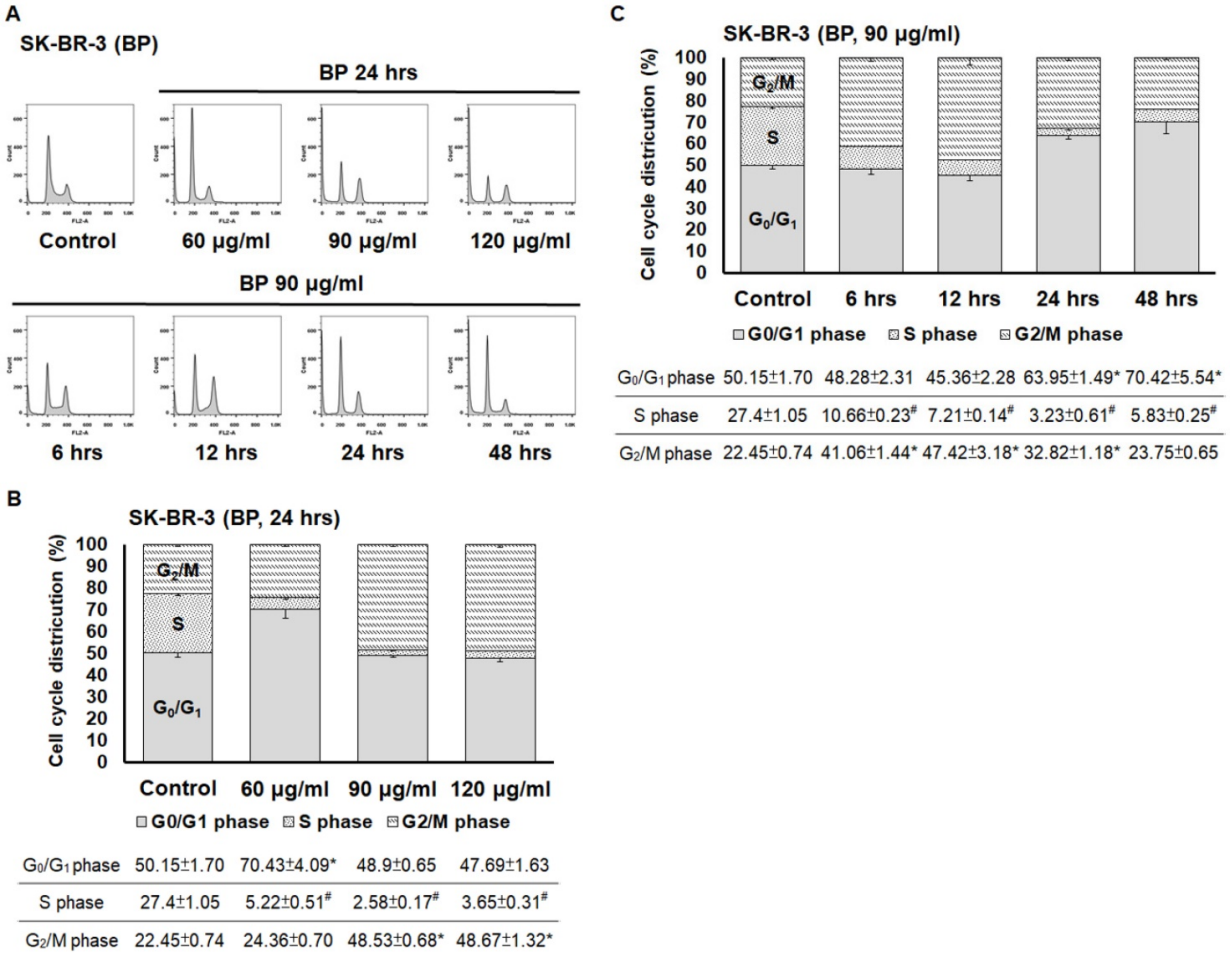
** BP induced cell cycle arrest at G_0_/G_1_ phase in breast cancer cells.** Cells (2×10^6^) were exposed to BP (60, 90 and 120 μg/ml) for different time points and then harvested and analyzed using flow cytometry. (A) The peak of cell cycle distribution; (B) Quantifiable cell cycle distribution with different doses of BP; (C) Quantifiable cell cycle distribution with different time points of BP. *: Indicated a significant increase between BP/LPPC or BP and control (*P* < 0.05). #: Indicated a significant decrease between BP/LPPC or BP and control (*P* < 0.05).

**Figure 7:**
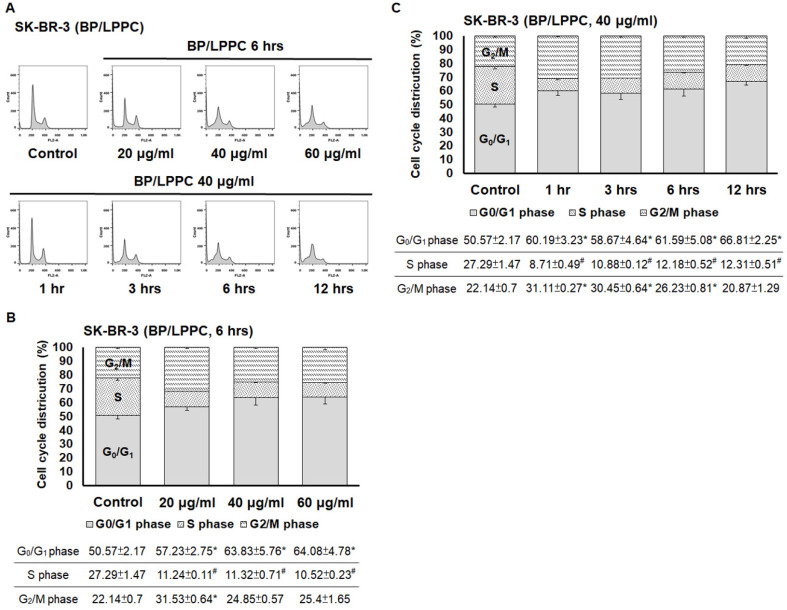
** BP/LPPC induced cell cycle arrest at G_0_/G_1_ phase in breast cancer cells.** Cells (2×10^6^) were exposed to BP/LPPC (20, 40 and 60 μg/ml) for different time points and then harvested and analyzed using flow cytometry. (A) The peak of cell cycle distribution; (B) Quantifiable cell cycle distribution with different doses of BP/LPPC; (C) Quantifiable cell cycle distribution with different time points of BP/LPPC. *: Indicated a significant increase between BP/LPPC or BP and control (*P* < 0.05). #: Indicated a significant decrease between BP/LPPC or BP and control (*P* < 0.05).

**Figure 8:**
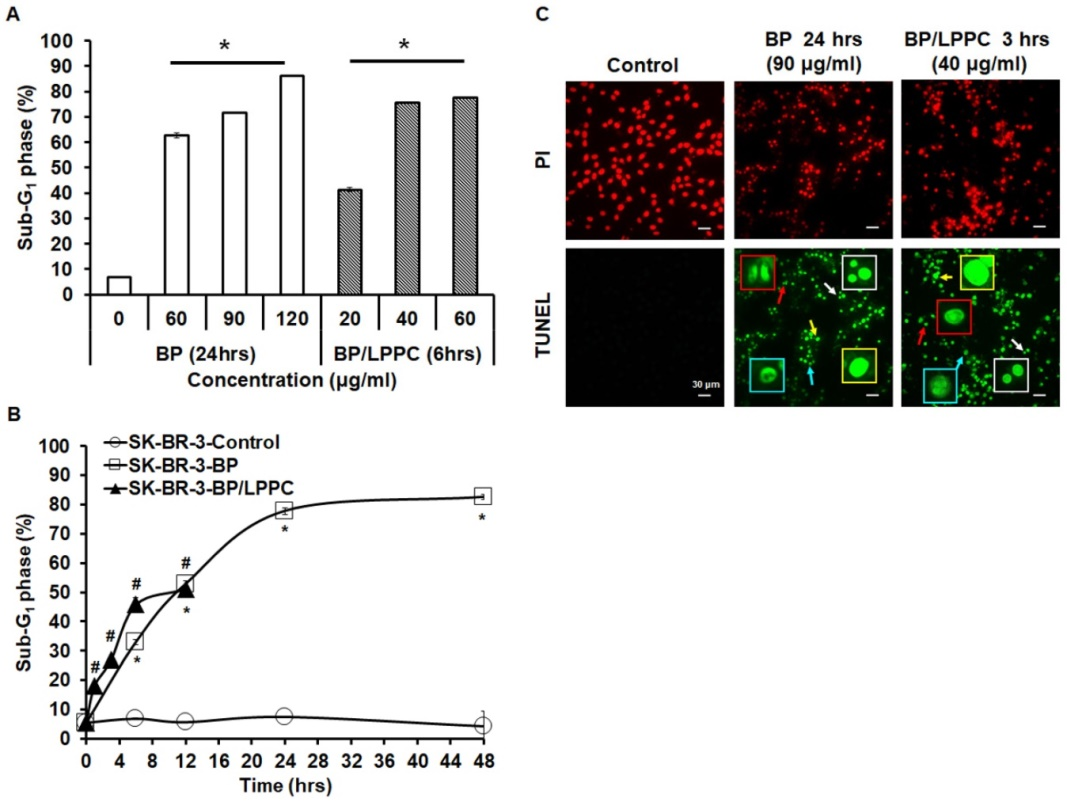
** BP/LPPC induced cell apoptosis in breast cancer cells.** Cells were exposed to different concentrations of BP or BP/LPPC for the indicated time points. (A, B) After treatment, cells were harvested and the sub-G_1_ phase was analyzed using flow cytometry. (C) Cells were harvested and stained with TUNEL (green) and PI (red) solution, and the cell death morphology was observed under microscopy in x400 field. Red arrow: apoptotic bodies; yellow arrow: DNA fragments; blue arrow: chromatin condensation; white arrow: anoikis. *: Indicated a significant difference between treatment and control groups (*P* < 0.05). *, #: Indicated a significant difference between BP (*) or BP/LPPC (#) treatment, respectively, and the control group (*P* < 0.05).

Next, we examined the effects of BP and BP/LPPC on cell cycle progression using flow cytometry. After exposure to BP (60, 90 and 120 μg/ml), the cell population of G_0_/G_1_ phase was increased in SK-BR-3 cells, and the cell population of G_2_/M phase was increased at a high dose of BP (Figure 6A and B). In addition, within 48 hours of BP treatment, the cell cycle arrest at G_0_/G_1_ phase along with the percentage of G_2_/M phase increased at 6-12 hours (Figure 6C). The results indicated that BP induced cell cycle arrest at G_0_/G_1_ phase (Figure 6). Subsequently, the cells were treated with BP/LPPC, and the results showed that BP/LPPC similarly induced cell cycle arrest at G_0_/G_1_ phase in a dose- and time-dependent manner, along with a slight percentage increase at G_2_/M phase (Figure 7). The results revealed that both BP and BP/LPPC blocked cell cycle progression at G_0_/G_1_ phase. It is noteworthy that after BP/LPPC or BP treatment, cells began accumulating in the sub-G_1_ phase, with the percentage at sub-G_1_ phase increasing in a dose- and time-dependent manner (Figure 8A and B). Consequently, to further investigate whether BP induced apoptosis in SK-BR-3 cells, we observed that BP/LPPC and BP induced apoptosis, resulting in the formation of apoptotic morphology, including DNA fragments, apoptotic bodies, and DNA condensation (Figure 8C). Taken together, BP and BP/LPPC not only mainly induced cell cycle arrest at G_0_/G_1_ phase but also triggered cell apoptosis in SK-BR-3 cells.

### BP/LPPC and BP modulated the expression of cell cycle regulators and induced apoptosis through activation of caspase cascade

**Figure 9:**
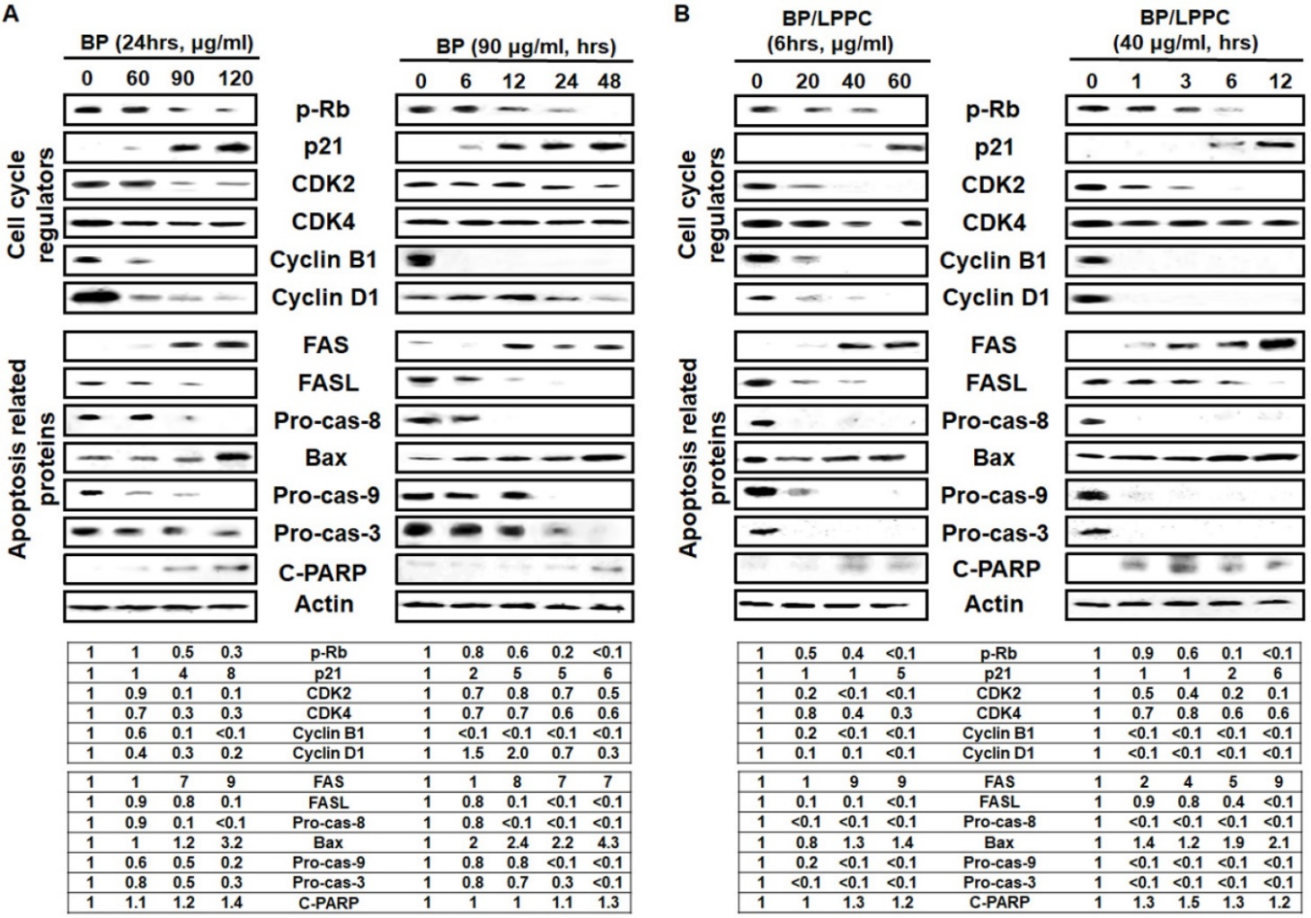
** BP and BP/LPPC induced cell cycle-related proteins and activated apoptosis proteins in breast cancer cells.** Proteins were extracted by RIPA lysis, resolved by sodium dodecyl sulfate-polyacrylamide gel electrophoresis and transferred onto PVDF. Afterward, membranes incubated primary antibodies overnight, second antibodies for 2 hrs and HRP at RT for 2 hrs, separately. The membranes were detected by a Luminescent Image Analyzer. (A) The cell cycle regulators and apoptosis-related proteins of BP; (B) The cell cycle regulators and apoptosis-related proteins of BP/LPPC.

To understand the molecular events associated with BP/LPPC and BP, and induced cell cycle was examined by Western blot. After BP/LPPC or BP treatment, the level of pRb, a tumor suppressor, was decreased, suggesting that dephosphorylation was reduced. The level of p21 was increased, and the cell cycle regulators, including cdk2, cdk4, cyclin B1, and cyclin D1, were decreased in a dose- and time-dependent manner (Figure 9). The mechanism of BP and BP/LPPC treatment to induce cell cycle arrest might be similar, indicating that LPPC encapsulation has no effect on the anti-proliferation effect of BP. Considering that the sub-G_1_ phase showed an increase with BP and BP/LPPC treatment, the next step was to delineate the signaling pathways by which BP/LPPC and BP induced apoptosis in SK-BR-3 cells. Both BP and BP/LPPC activated extrinsic apoptosis, followed by increasing the levels of FAS and decreasing FASL and procaspase-8. Conversely, BP and BP/LPPC also activated intrinsic apoptosis, along with increasing the level of Bax and decreasing procaspse-9, and finally, downstream, the level of procaspase-3 was decreased and increased cleaved PARP, which turned on the caspase cascade (Figure 9). The results suggested that BP/LPPC and BP activated extrinsic and intrinsic apoptosis pathways in a dose- and time-dependent manner. Therefore, these results demonstrated that BP presented inhibitory effects on SK-BR-3 cells via modulation of cell cycle-related proteins to restrain cell cycle and mediation of caspase cascade progression to improve cell apoptosis. Moreover, LPPC encapsulation not only maintained the inhibitory effects of BP, but also improved the anti-cancer effects on SK-BR-3 cells.

### BP/LPPC exhibited synergistic effects with doxorubicin in SK-BR-3 and MDA-MB-231 cells

**Figure 10:**
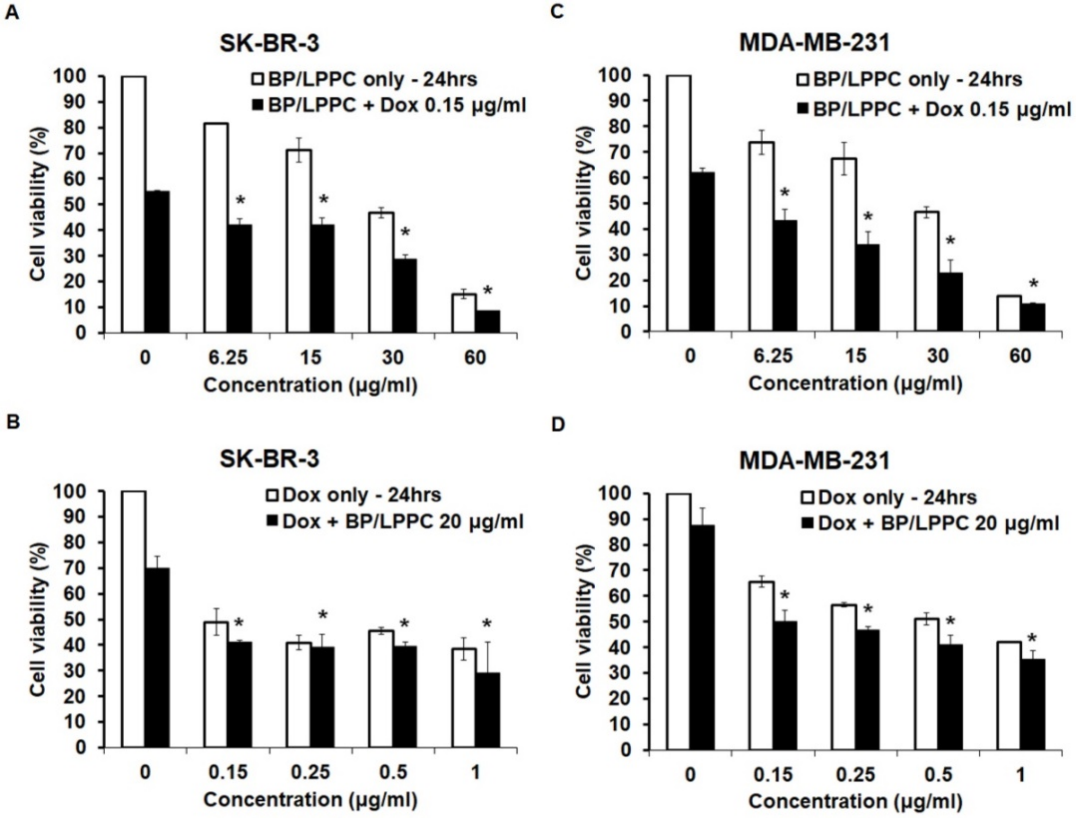
** BP/LPPC combined with doxorubicin had a synergistic effect on breast cancer cells.** SK-BR-3 and MDA-MB-231 cells were treated with BP/LPPC combined with doxorubicin. (A, B) SK-BR-3 cells. (C, D) MDA-MB-231 cells. *: Indicated a significant difference between the combination of drugs and drug alone (black bar) (*P* < 0.05).

We investigated the synergistic effect of BP/LPPC and doxorubicin to reduce the dose of doxorubicin and diminish its side effect, and the results showed that the inhibitory effects of doxorubicin combined with different concentrations of BP/LPPC increased the cytotoxic effects in a dose-dependent manner in both cell types (Figure 10A and C). However, when different concentrations of doxorubicin were combined with BP/LPPC, the concentration did not lead to remarkable toxicity in either cell type at 24 hours (Figure 10B and D). The combination index of doxorubicin and BP/LPPC was 0.75 and 0.41 in SK-BR-3 and MDA-MB-231 cells, respectively. These data demonstrated that doxorubicin combined with BP/LPPC had a synergistic effect on reducing both cell growths.

## Discussion

Breast cancer has a high incidence rate and is increasing yearly worldwide, especially in women. BP is commonly used in the treatment for some gynecological diseases and is well-known for its anti-cancer ability against several cancer types through cell cycle arrest or cell apoptosis. Moreover, BP has found the anticancer activity in MDA-MB-231 and MCF-7 cells through induction of cell apoptosis, block of cell cycle progression, the contribution of DNA damage and repression of cell migration and invasion *in vitro* 24. However, the anti-cancer mechanisms in SK-BR-3 cells remained unclear until our results presented that BP blocked the cell cycle at the G_0_/G_1_ phase and induced both intrinsic and extrinsic apoptosis pathways. In this study, we first demonstrated that BP presented inhibitory effects on SK-BR-3 cells by induction of cell apoptosis and block of cell cycle progression, and the results showed that SK-BR-3 cells were sensitive to BP; by contrast, MDA-MB-231 cells were resistant to BP. The mechanisms of BP-induced cell cycle arrest were through the reduction of pRb to block the E2F factor recruitment and increasing p21 protein expression to inhibit the associated cyclin/CDK protein expression. Afterward, BP was found to induce the extrinsic apoptotic pathway via FASL binding to FAS to activate the cleavage of procaspase-8. Moreover, Bax protein expression increased, which suggested that BP might affect mitochondrial function and lead to the induction of the intrinsic apoptotic pathway. The results revealed that BP induced intrinsic as well as extrinsic apoptosis pathway and was similar to previous research had found in MDA-MB-231 cells 24.

Although BP showed a good inhibitory effect on breast cancer cells, in consideration of the property of BP having some disadvantages, LPPC was used to overcome the drawbacks, such as poor water solubility and instability in conditional environments. In view of, the application and previous studies of LPPC provide strong supportive evidence on the encapsulation capacity and anticancer capacity in breast cancer therapy. Additionally, there is no evidence showing that BP/LPPC could inhibit breast cancer growth in either triple-positive breast cancer cells (SK-BR-3) or triple-negative breast cancer cells (MDA-MB-231). Herein, in the present study, our results indicated that after LPPC encapsulation, the anti-breast cancer activity of BP maintained its cytotoxicity in water and protein-rich environments. Moreover, LPPC encapsulation remained the BP activity and accelerated the amount of BP entering that increased cytotoxicity of BP to triple and positive breast cancer cells. Comparison with BP/Lipo which passive target cells, BP/LPPC showed better inhibitory effects on breast cancer cells. Interestingly, the IC_50_ values of BP/Lipo to MCF-7, BCM-1, CMT-1 and JC cells were much higher than that of BP. In the methodological view, the cytotoxicity of BP in BP/Lipo might be decreased its activities during the manufacturing process despite liposome encapsulation and different cell characteristics may be one of the reasons to affect the IC_50_ values. In the results, we also found that lots of BP/LPPC were rapidly entrapped into SK-BR-3 cells within 15 minutes and continuously for 60 minutes, significantly, which might be the reason why BP/LPPC contributed to the higher cytotoxicity to breast cancer cells. Therefore, LPPC encapsulation not only improved the uptake of BP but also amplified the cell cytotoxic effects of BP, which induced more cell death in BP/LPPC treatment. It is known that liposome is passive targeting to tumor cells and LPPC with positive charge might induce cell internalization. As a result, we first revealed that LPPC induced cell endocytotic pathways in breast cancer cells, such as clathrin-mediated, caveolae-mediated, and clathrin or caveolae independent pathway, to accelerate BP entrapment into the cytoplasm, leading to serial effects. According to the results, we assumed that BP/LPPC might mainly activate the caveolae-mediated pathway to internalize BP into cytoplasm because the content of BP dramatically decreasing after treatment with FIII inhibitor. Besides, researches reveal that caveolae are 50-100 nm Ω-shaped microdomains that compose of cholesterol and scaffold proteins named caveolins that inclusive of caveolin-1, caveolin-2, and caveolin-3. Caveolin-1 plays various roles in endocytosis, signal transduction, cell proliferation, apoptosis, and metastasis 42. Moreover, caveolin-1 is highly expressed in MDA-MB-231 cells to facilitate the drug-resistant signal transduction and moderately expressed in SK-BR-3 cells to affect the chemosensitivity to chemodrugs 43. Hence, after LPPC encapsulation, it can accelerate cell uptake via a caveolae-mediated pathway to fast induce cell apoptosis and reduce the potential of drug-resistant generation. Next, a comparison of anti-breast cancer effects between BP and BP/LPPC at different time points and the Western blot results revealed that BP/LPPC markedly affects protein expressions at 1-3 hours; however, BP possessed anti-breast cancer effects at 6-12 hours. These results suggested that LPPC encapsulation would effectively accelerate and enhance the anti-cancer effects of BP on blocking the cell cycle process and inducing cell apoptosis *in vitro*.

Nevertheless, we demonstrated the inhibitory effect of BP/LPPC on breast cancer *in vitro*, its detailed inhibitory effect and mechanisms in tumor-bearing mice is a weakness in the present study. To supportive the anticancer activity of BP/LPPC *in vivo*, we would descript our previous study that revealed BP/LPPC suppressed GBM growth in xenograft and orthotopic animal model and, therefore, we assumed the same formulation of BP/LPPC was implied similar anti-cancer effects in breast cancer *in vivo* 36. Furthermore, in our previous research, we utilized LPPC to encapsulate doxorubicin and randomly adsorbed Herceptin on the surface of LPPC. The results indicated that Herceptin adsorbed on LPPC directed towards HER2/neu‑positive cells within 12 hours to induce increasing of cleaved caspase-3 expression and suppress the level of PCNA, VEGF, VEGFR-1, and VEGFR-2 *in vivo* 34. The LPPC‑delivery system provided a better therapeutic efficacy in a xenografted model. In view of previous results, we wonder whether BP/LPPC combined with doxorubicin had synergistic effects to reduce the dose of doxorubicin and diminish its side effect and our results revealed a synergistic effect of BP/LPPC and doxorubicin *in vitro*. In conclusion, BP and BP/LPPC induced cytotoxic activity through cell cycle arrest and cell apoptosis. Moreover, LPPC encapsulation not only enhanced the cytotoxic activity but also rapidly induced the death of cells that did not change the anti-cancer activity of BP. Therefore, BP and BP/LPPC might be potential therapeutic drugs or functional food in the future.

## Figures and Tables

**Table 1:** Growth inhibition concentration (IC_50_) of BP, BP/LPPC, BP/Lipo and Dox

Cell line	Tumor type	BP (μg/ml)	BP/LPPC (μg/ml)	BP/Lipo (μg/ml)	Dox (μg/ml)	Fold
**Breast cancer**						
SK-BR-3	Human breast adenocarcinoma	74.1±0.7	22.9±5.6 ^a, b, c, d^	65.3±0.9	1.12±1.8	3.23
MDA-MB-231	Human breast adenocarcinoma	132.0±3.6	26.8±2.2 ^a, b, c, d^	147.3±3.8	> 2	4.93
MCF-7	Human breast adenocarcinoma	140.8±0.4	33.3±0.2^ a, b, c^	154.3±9.2	ND	4.23
BCM-1	Human breast carcinoma	50.9±0.2	15.9±4.0 ^a, b, c^	184.6±4.2	ND	3.19
CMT-1	Canine mammary gland tumor	93.1±4.9	25.7±9.3^ a, b, c^	142.6±7.7	ND	3.63
CF41	Canine breast cancer	153.5±0.7	34.6±3.5^ a, b, c^	115.0±5.3	ND	4.44
JC	Mouse breast adenocarcinoma	38.3±3.3	14.7±2.0^ a, b, c^	88.4±0.9	ND	2.60
**Normal cell**						
SVEC	Mouse endothelial cell	158.9±0.8	47.9±4.1	138.3±5.7	ND	3.31

**Notes:** Values were presented as the mean ± SD (μg/ml) at 24 hrs. a: Indicated a significant difference compared to the BP treatment group. b: Indicated a significant difference compared to the Bp/Lipo treatment group. c: Indicated a significant difference compared to SVEC cells. d: Indicated a significant difference compared to the Dox treatment group (*P* < 0.05). Fold: Indicated the IC_50_ values of BP/ the IC_50_ values of BP/LPPC. Abbreviations: Dox: doxorubicin. Fold: comparison of BP with BP/LPPC.

**Table 2:** The selective index between JC and SVEC cells

Normal cells (IC_50_)/ Tumor cells (IC_50_)	BP	BP/LPPC	BP/Lipo
SVEC/JC	4.15	3.23	1.56

Note: Selectivity index (SI) = IC_50_ of normal cells/IC_50_ of JC cells. SI > 2: indicated that drugs have good selectivity; SI < 2: indicated that drugs have poor selectivity.
